# Highly efficient Fenton and enzyme-mimetic activities of NH_2_-MIL-88B(Fe) metal organic framework for methylene blue degradation

**DOI:** 10.1038/s41598-018-23557-2

**Published:** 2018-03-26

**Authors:** Jianchuan He, Yao Zhang, Xiaodan Zhang, Yuming Huang

**Affiliations:** 1 The Key Laboratory of Eco-environments in Three Gorges Reservoir Region, Ministry of Education, College of Chemistry and Chemical Engineering, Southwest University, Chongqing, 400715 PR China; 2 School of Basic Medical Sciences, North Sichuan Medical College, Nanchong, 637000 PR China

## Abstract

Here, we show that NH_2_-MIL-88B(Fe) can be used as a peroxidase-like catalyst for Fenton-like degradation of methylene blue (MB) in water. The iron-based NH_2_-MIL-88B(Fe) metal organic framework (MOF) was synthesized by a facile and rapid microwave heating method. It was characterized by scanning electron microscopy, Fourier transform infrared spectroscopy, powder X-ray diffraction, and the Brunauer–Emmett–Teller method. The NH_2_-MIL-88B(Fe) MOF possesses intrinsic oxidase-like and peroxidase-like activities. The reaction parameters that affect MB degradation were investigated, including the solution pH, NH_2_-MIL-88B(Fe) MOF and H_2_O_2_ concentrations, and temperature. The results show that the NH_2_-MIL-88B(Fe) MOF exhibits a wide working pH range (pH 3.0–11.0), temperature tolerance, and good recyclability for MB removal. Under the optimal conditions, complete removal of MB was achieved within 45 min. In addition, removal of MB was above 80% after five cycles, showing the good recyclability of NH_2_-MIL-88B(Fe). The NH_2_-MIL-88B(Fe) MOF has the features of easy preparation, high efficiency, and good recyclability for MB removal in a wide pH range. Electron spin resonance and fluorescence probe results suggest the involvement of hydroxyl radicals in MB degradation. These findings provide new insight into the application of high-efficient MOF-based Fenton-like catalysts for water purification.

## Introduction

Metal organic frameworks (MOFs) are composed of metal ions with organic linkers. They have received great attention because of their peculiar structural properties, such as large specific surface areas, tuneable porosities, good thermal stabilities, and uniform structured nanoscale cavities. Various MOF structures have been designed and synthesized for a wide variety of applications, including separation, storage of molecules, sensors, luminescence, and catalysis^1–6^, among which chemical catalysis is particularly interesting^6–10^. However, investigation of the catalytic applications of MOFs has mainly focused on model organic reactions^7–10^. Recently, several MOFs, including Fe-containing MOFs (MIL-101(Fe), MIL-53(Fe), MIL-88B(Fe), (Me_3_Sn)_4_Fe(CN)_6_, and MIL-53(Fe)), have been investigated as photocatalysts for CO_2_ reduction^11^, Cr(VI) reduction^12^, and reactive dye degradation^13–17^. In addition, MIL-100(Fe) and Fe^II^@MIL-100(Fe) have been used as Fenton-like catalysts for removal of azo-dye from wastewater^18^. However, low pH (pH 3.0) is needed to obtain high catalytic performance, and total organic carbon (TOC) removal by Fe^II^@MIL-100(Fe) in the first 8 h is low (13–29%)^18^. Zhao *et al*.^19^ found that MOF(2Fe/Co)/carbon aerogel shows good electrocatalytic and photocatalytic activities. They showed that it can be used to design a solar photo-electro-Fenton process for removal of rhodamine B and dimethyl phthalate at the optimal pH of 3.0. Li *et al*.^20^ reported an Fe(II) bipyridinedicarboxylate based MOF as a Fenton catalyst for degradation of phenol in the narrow pH range of 3–6. Martínez *et al*.^21^ reported that Fe-BTC and MIL-100(Fe) can be used as Fenton catalysts for MB removal with TOC reduction of about 55% in 60 min at an initial pH of 4. However, the limited operational pH range and the need for acidic conditions (pH 3.0–4.0) for the degradation reaction (i.e., the water must be acidified) in the above studies are drawbacks that hinder the use of the MOFs in water purification. Most recently, MOFs have been reported to exhibit peroxidase-like catalytic activity^22–26^. This opens the door for development of MOF-based nanoscale platforms for sensing application in the bioanalytical field^27,28^. However, research of MOFs as enzyme mimetics in an aqueous environment is rare. The first member of enzyme-like active MOFs is PCN-222(Fe) with porphyrinic Fe(III) centers^22^. Subsequently, MIL-53(Fe), MIL-68(Fe), and MIL-100(Fe) with intrinsic peroxidase-like catalytic activity have been developed^23–26^. Application of these active MOFs as peroxidase mimetics for colorimetric biosensing has also been proposed. These studies demonstrate the potential of MOFs as mimic enzymes. However, application of MOF-based mimic enzymes is still limited. There have been no reports of using MOFs as mimic enzymes with wide pH tolerance and good recyclability for degradation of toxic dyes in water by a Fenton-like reaction.

Herein, we report removal of methylene blue (MB) by Fe-based MOFs (i.e., NH_2_-MIL-88B(Fe) MOF) to evaluate the potential of using MOFs as peroxidase mimic catalysts in the Fenton-like reaction for removal of organic dyes from contaminated water. The NH_2_-MIL-88B(Fe) MOF was synthesized by a fast and facile microwave-assisted approach^29^. Interestingly, the as-prepared NH_2_-MIL-88B(Fe) MOF shows intrinsic oxidase-like activity, peroxidase-like activity, and excellent catalytic performance for MB degradation by a Fenton-like reaction in a wide pH range. The narrow pH range remains a limitation for removal of organic pollutants from water using Fenton-based reactions^30,31^. The catalytic mechanism of NH_2_-MIL-88B(Fe) was investigated and a possible mechanism is proposed based on electron spin resonance (ESR) and fluorescence probe results for detection of ∙OH free radicals, which confirms the major role of ∙OH free radicals in degradation of MB. The adsorption isotherm for adsorption of MB on NH_2_-MIL-88B(Fe) was determined and the adsorption kinetics was investigated. The effects of NH_2_-MIL-88B(Fe) MOF and H_2_O_2_ concentrations, solution pH, and reaction temperature were also investigated based on the MB and TOC removal efficiencies. Finally, the reusability of NH_2_-MIL-88B(Fe) was investigated. The results show the rapid and good recyclability of NH_2_-MIL-88B(Fe) for removal of MB from wastewater in a wide pH range by a Fenton-like reaction.

## Results and Discussion

### Characterization and enzyme-like activity of NH_2_-MIL-88B(Fe)

**Figure 1:**
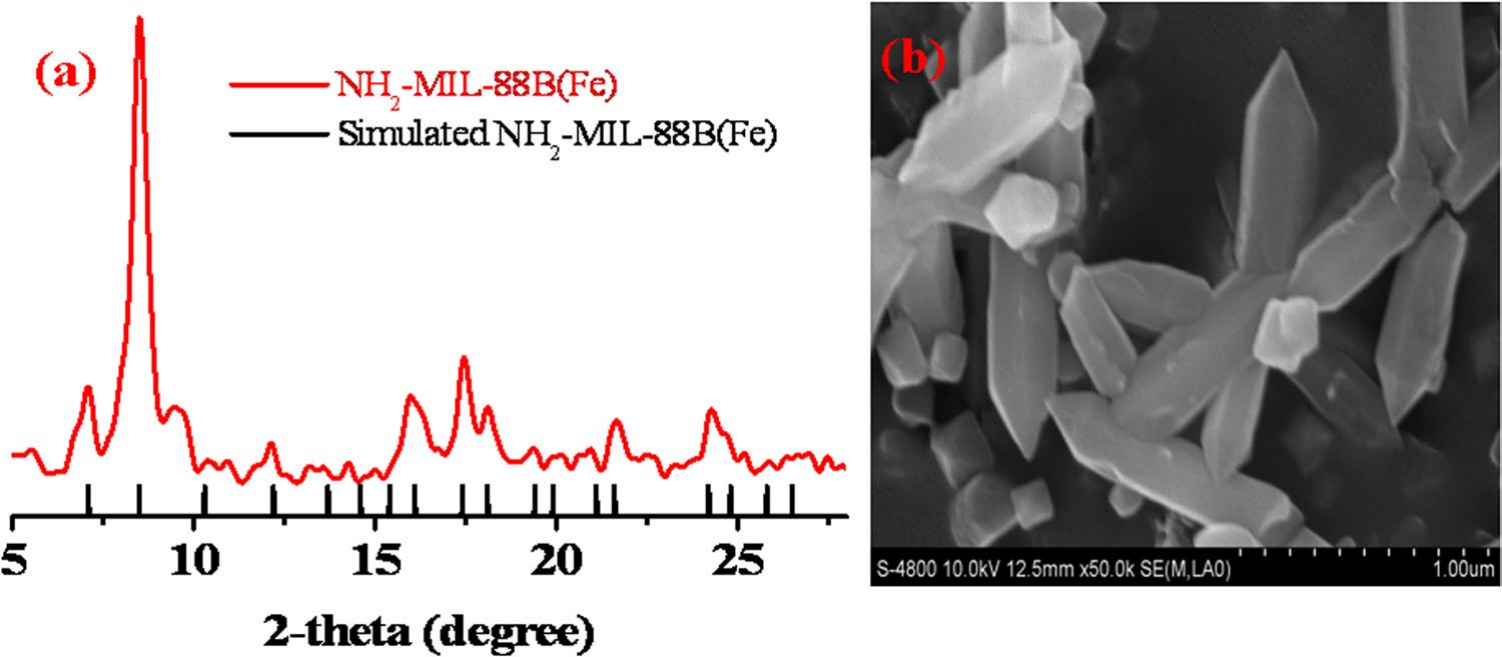
(**a**) The PXRD patterns of the as-prepared NH_2_-MIL-88B(Fe) (red line) and the simulated patterns of the NH_2_-MIL-88B(Fe) (black short bars). (**b**) SEM image of the as-prepared NH_2_-MIL-88B(Fe).

NH_2_-MIL-88B(Fe) was synthesized by a microwave-assisted approach, which has attracted growing attention as a promising way to synthesize MOF materials^29,32–37^. It has the advantages of speed, high efficiency, and high yield^29,32^. However, the microwave-assisted approach has rarely been applied for synthesis of NH_2_-MIL-88B(Fe)^29^. In this work, the NH_2_-MIL-88B(Fe) MOF was synthesized by microwave heating with a yield of ca. 30%. The crystal structure of the as-prepared material was confirmed to be NH_2_-MIL-88B(Fe) by powder X-ray diffraction (PXRD) (Fig. 1a), which is in agreement with a previous report^29^ and indicates the success of NH_2_-MIL-88B (Fe) synthesis. It is noted that the PXRD pattern of NH_2_-MIL-88B(Fe) in this work is different from that in Shi’s work^12^. This could be caused by the difference in the solvents used for treatment of as-prepared NH_2_-MIL-88B(Fe)^29^. Similar to previous work, the scanning electron microscopy (SEM) image shows that the synthesized NH_2_-MIL-88B(Fe) has a needle-shaped morphology^29^ with needle sizes of approximately 800 nm in length and 300 nm in diameter (Fig. 1b). Successful formation of NH_2_-MIL-88B(Fe) was further confirmed by Fourier transform infrared (FTIR) spectroscopy (Fig. S1, Supporting Information). The characteristic absorption peaks associated with NH_2_-MIL-88B(Fe) are observed at around 3490 and 3370 cm^−1^, corresponding to the symmetric and asymmetric stretching vibrations of the primary amine groups. The peak at around 1700 cm^−1^ is attributed to the carboxylic group. The FTIR spectrum is the same as those of previous studies^24,37^. The specific surface area of NH_2_-MIL-88B(Fe) MOF was calculated to be 163.9 m^2^/g.

**Figure 2:**
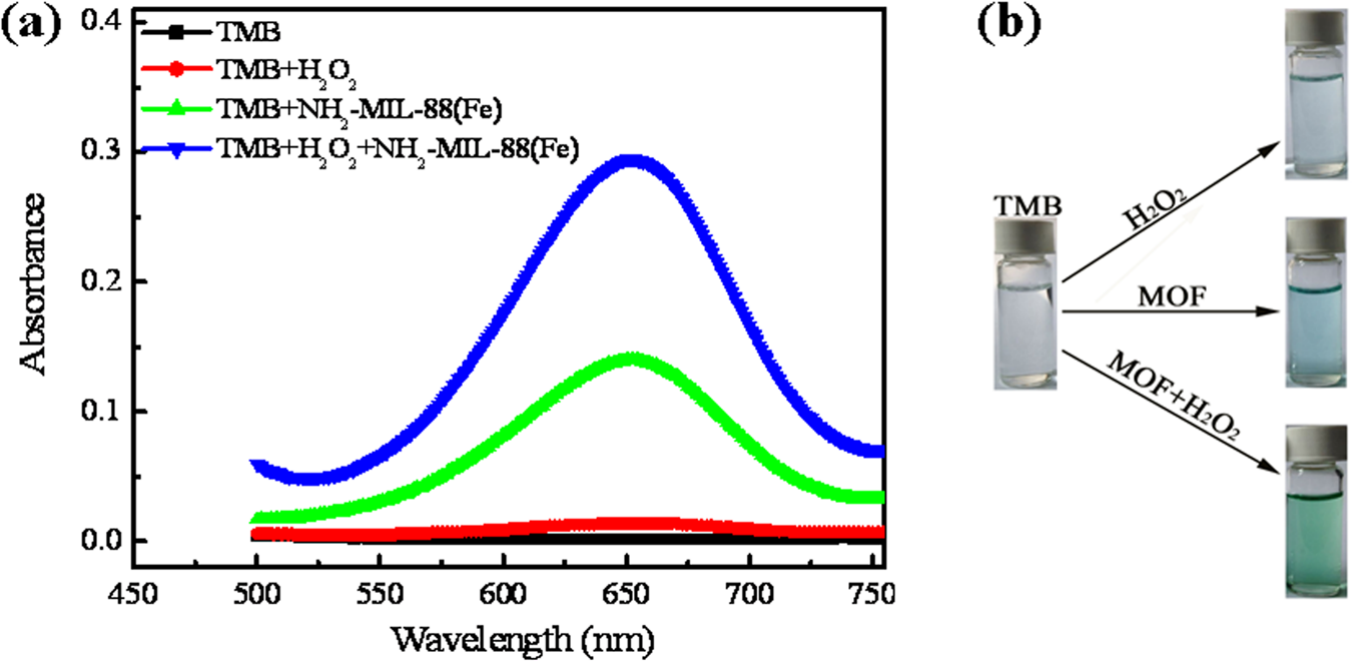
(**a**) UV/vis spectroscopy of 0.05 mM TMB after 20-min reaction with and without 0.1 mM H_2_O_2_ in 0.2 M acetate buffer (pH 4.0) in the absence and presence of 40 mg/L NH_2_-MIL-88B(Fe). (**b**) Images colour reaction of TMB oxidation by H_2_O_2_ or dissolved O_2_ in the absence and presence of NH_2_-MIL-88B(Fe).

Before using NH_2_-MIL-88B(Fe) in the Fenton-like reaction, the enzyme-like activity of NH_2_-MIL-88B(Fe) was evaluated by oxidizing the typical peroxidase substrate 3,3′,5,5′-tetramethylbenzidine (TMB) in the presence and absence of H_2_O_2_. As shown in Fig. 2a, similar to other enzyme mimic reactions, the typical absorbance peak of the oxidation product of TMB is located at 652 nm^38,39^. The absorbance of the peak at 652 nm significantly increases in the presence of NH_2_-MIL-88B(Fe), confirming the peroxidase-like activity of NH_2_-MIL-88B(Fe). Interestingly, even in the absence of H_2_O_2_, NH_2_-MIL-88B(Fe) can catalyze oxidation of TMB by dissolved O_2_ to produce the typical colour reaction (Fig. 2b), indicating that NH_2_-MIL-88B(Fe) also exhibits oxidase-like activity. Thus, NH_2_-MIL-88B(Fe) can potentially be used as an enzyme mimic catalyst in the Fenton-like reaction for removal of organic pollutants in the presence of H_2_O_2_.

### Role of NH_2_-MIL-88B(Fe) in Fenton-like removal of MB dye

**Figure 3:**
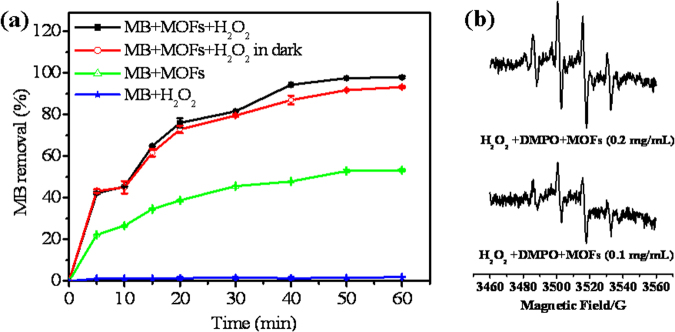
(**a**) MB removal versus time in the absence and presence of H_2_O_2_ and NH_2_-MIL-88B(Fe) on different conditions. Reaction conditions: pH 5.6, 0.2 g/L of NH_2_-MIL-88B(Fe), 0.2 M of H_2_O_2_, 20 mg/L of MB. Error bars represent one standard deviation for three measurements. (**b**) The effect of the concentration of NH_2_-MIL-88B(Fe) on the hydroxyl radical formation in the H_2_O_2_-DMPO system.

As-synthesized NH_2_-MIL-88B(Fe) was applied as a Fenton-like catalyst for removal of MB as a model organic dye. Figure 3a shows the kinetics of MB removal in the presence and absence of H_2_O_2_ and NH_2_-MIL-88B(Fe). Slight removal of MB occurs in the absence of NH_2_-MIL-88B(Fe), indicating that direct oxidation of MB by H_2_O_2_ is limited. In contrast, when only the NH_2_-MIL-88B(Fe) catalyst is present in the MB solution, the removal efficiency increases with increasing reaction time. The peak removal efficiency of about 52% occurs at 50 min, which is mainly caused by adsorption of MB on NH_2_-MIL-88B(Fe). Notably, when both NH_2_-MIL-88B(Fe) and H_2_O_2_ are present in the solution of MB, almost 100% removal of MB is achieved after 50 min. The above observation confirms that about half of the MB removal is attributed to NH_2_-MIL-88B(Fe) acting as a strong peroxidase mimic in aqueous media. This is probably because degradation of MB by H_2_O_2_ in the presence of NH_2_-MIL-88B(Fe) mainly originates from generation of highly active ∙OH free radicals. The presence of ∙OH free radicals in the catalytic process was confirmed by ESR spectroscopy. The ESR spectrum in the presence of NH_2_-MIL-88B(Fe) shows the four-fold characteristic peak of the typical DMPO–∙OH adduct with an intensity ratio of 1:2:2:1 (Fig. 3b), suggesting that NH_2_-MIL-88B(Fe) can decompose H_2_O_2_ to ∙OH radicals. Furthermore, the intensity of the characteristic peak increases with increasing NH_2_-MIL-88B(Fe) concentration (Fig. 3b), confirming the catalytic ability of NH_2_-MIL-88B(Fe) to decompose H_2_O_2_ to ∙OH radicals. MOFs have previously been used as photocatalysts to degrade active dyes^13–17^. Interestingly, as a control, when the mixture of NH_2_-MIL-88B(Fe), MB, and H_2_O_2_ was placed in the dark, there was no significant change in MB removal (Fig. 3a). This suggests that NH_2_-MIL-88B(Fe) shows high catalytic activity for MB removal without the need for light irradiation, further confirming the peroxidase-like activity of NH_2_-MIL-88B(Fe). Hence, NH_2_-MIL-88B(Fe) can be used as an effective peroxidase mimic catalyst for removal of MB without the need for light irradiation.

Recently, MOFs have been used for removal of hazardous organic compounds, such as benzene^36^, dyes^40–45^, nitrobenzene^46,47^, pharmaceuticals and personal care products^48,49^, phenolic compounds^50,51^, aniline^51^, herbicides^52^, and organoarsenic compounds^53^, form aqueous media for water purification. This demonstrates that MOFs are promising materials for organic pollution clean-up. In our case, a significant fraction of MB (52%) was removed by direct adsorption to NH_2_-MIL-88B(Fe). To understand the adsorption performance, the time-dependent adsorption capacity was determined to investigate the kinetics of adsorption of MB on NH_2_-MIL-88B(Fe). The kinetics of MB adsorption on NH_2_-MIL-88B(Fe) shows a rapid initial uptake stage and a subsequent stable stage (Fig. S2, Supporting Information), indicating rapid adsorption of MB on NH_2_-MIL-88B(Fe). The pseudo-first-order and pseudo-second-order equations were used to fit the adsorption kinetics data (Supporting Information). The correlation coefficient for the pseudo-second-order kinetic model is high (>0.99) (Table S1, Supporting Information). The pseudo-second-order model fits the experimental data better than the pseudo-first-order model (Table S1, Supporting Information). Similar results have been obtained for adsorption of MB on MOF-235^41^ and methyl orange on Cr-BDCs, such as MIL-53 and MIL-101^42^. Interestingly, the kinetic constant for MB adsorption on NH_2_-MIL-88B(Fe) is larger than that for adsorption of MB on MOF-235^41^ (Table S2, Supporting Information), confirming the fast removal rate of MB by NH_2_-MIL-88B(Fe).

The adsorption isotherm was determined after adsorption/desorption equilibrium for 1 h (Fig. S2, Supporting Information). The adsorption isotherm of MB on NH_2_-MIL-88B(Fe) is shown in Fig. S3 (Supporting Information). The Langmuir and Freundlich models were used to describe adsorption of MB on NH_2_-MIL-88B(Fe) (Supporting Information). The results show that the Langmuir model is suitable to describe adsorption of MB on NH_2_-MIL-88B(Fe) (Table S3, Supporting Information). The maximum adsorption capacity (*Q*_max_) of NH_2_-MIL-88B(Fe) for MB is 61.46 mg/g. This can be attributed to electrostatic interaction between the positive charge of MB and the negative charge of COO^−^, which is verified by the zeta potential of NH_2_-MIL-88B(Fe) (Fig. S4, Supporting Information). The surface charge of NH_2_-MIL-88B(Fe) remains negative. Thus, adsorption of positively charged MB on negatively charged NH_2_-MIL-88B(Fe) could be by electrostatic attraction. The charge-balancing anion of NH_2_-MIL-88B(Fe)^41^ and π–π interaction^54^ between the benzene rings of NH_2_-MIL-88B(Fe) MOF and MB could also be responsible for MB adsorption.

### Optimization of the experimental conditions for Fenton-like degradation of MB

**Figure 4:**
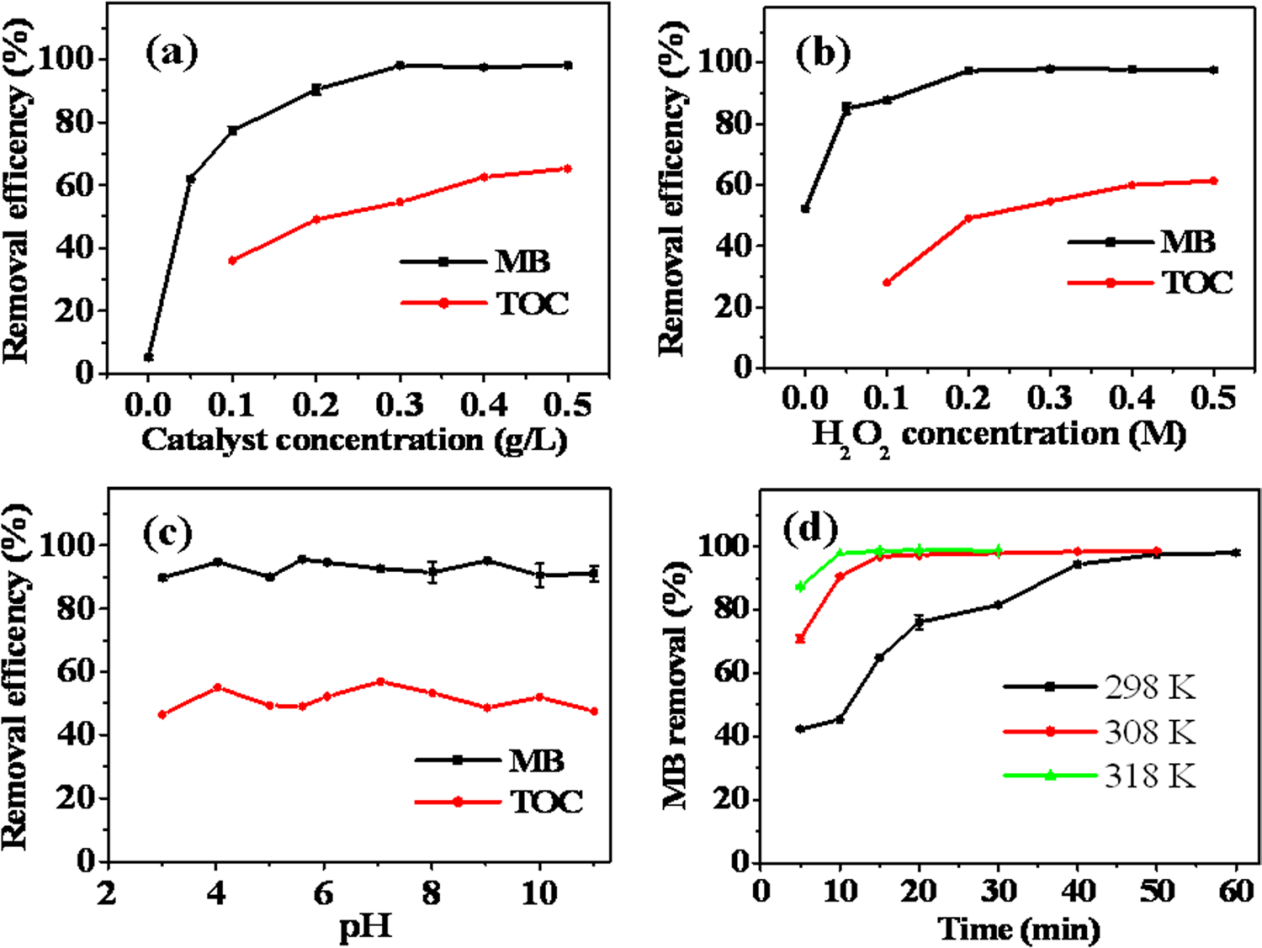
(**a**) Effect of catalyst concentration on the removal of MB and TOC (25 °C, pH 5.6, concentration of H_2_O_2:_ 0.2 M, concentration of initial MB: 20 mg/L). (**b**) Effect of H_2_O_2_ concentration on the removal of MB and TOC (25 °C, pH 5.6, catalyst concentration: 0.2 g/L, concentration of initial MB: 20 mg/L). (**c**) Effect of solution pH on the removal of MB and TOC (25 °C, catalyst concentration: 0.2 g/L, concentration of H_2_O_2_: 0.2 M, concentration of initial MB: 20 mg/L). (**d**) Effect of reaction temperature on the removal of MB (pH 5.6, catalyst concentration: 0.2 g/L, concentration of H_2_O_2_: 0.2 M, concentration of initial MB: 20 mg/L).

The effect of NH_2_-MIL-88B(Fe) concentration on MB degradation was investigated in the range 0–0.5 g/L, and the results are shown in Fig. 4a. The MB degradation efficiency increases from 5% to 90% with increasing NH_2_-MIL-88B(Fe) concentration from 0 to 0.2 g/L. This enhancement in the degradation efficiency is because of an increase in the ∙OH radicals (Fig. 3b). As the amount of NH_2_-MIL-88B(Fe) increases, more hydroxyl radicals are generated. However, with a further increase in the NH_2_-MIL-88B(Fe) concentration, there is only a slight change in the MB removal efficiency. It is interesting to note that TOC removal steadily increases with increasing NH_2_-MIL-88B(Fe) concentration from 0.1 to 0.5 g/L, and the maximum TOC removal of 65.1% occurs at a NH_2_-MIL-88B(Fe) concentration of 0.5 g/L. Obviously, the removal efficiency of TOC increases as the catalyst concentration increases, while that of MB gets plateau at 0.3 g/L NH_2_-MIL-88B(Fe). This is because the color removal or decolorization is ascribed to the destruction of the whole molecular or the chromophore destruction, while TOC removal is attributed to the mineralization of the organic pollutants. Hence, 100% color removal may not mean the completely mineralization of organic pollutants^55^. At a NH_2_-MIL-88B(Fe) concentration of 0.3 g/L, the color removal was as high as 97% above, while the TOC removal was only 54.6% (Fig. 4a). Thus, a further increase in catalyst dosage above 0.3 g/L caused no obvious decolorization of MB, although H_2_O_2_ decomposition was accelerated as the dose of NH_2_-MIL-88B(Fe) increased and more hydroxyl radicals generated (Fig. 3b). This suggests that some organic intermediates with low molecules were generated^56^ and incompletely mineralization of MB. Hence, TOC removal steadily increases with increasing NH_2_-MIL-88B(Fe) concentration. This rapid reduction in the TOC could be related to the enhanced generation of ∙OH radicals at a high concentration of NH_2_-MIL-88B(Fe). Considering that 90% MB removal efficiency and about 50% mineralization of MB are achieved at 0.2 g/L NH_2_-MIL-88B(Fe), 0.2 g/L was chosen as the NH_2_-MIL-88B(Fe) concentration in the subsequent experiments.

The effect of H_2_O_2_ concentration on the degradation efficiency of MB and TOC removal was investigated, and the results are shown in Fig. 4b. The experimental results show that the degradation efficiency of MB rapidly increases with increasing concentration of H_2_O_2_ in the range 0–0.2 M. However, there is only a slight change in MB removal when the concentration of H_2_O_2_ is above 0.2 M. TOC removal steadily increases with increasing H_2_O_2_ concentration in the range 0–0.5 M, and the maximum TOC removal of 61.3% occurs when the H_2_O_2_ concentration is 0.5 M. In this work, the optimized H_2_O_2_ concentration was set at 0.2 M.

The solution pH is an important factor that can remarkably affect catalytic reactions. Thus, the effect of pH on the degradation efficiency of MB and TOC removal was investigated in the pH range 3.0–11.0 (Fig. 4c). The pH has no significant influence on degradation of MB, and the MB removal efficiency is in the range 88.1–95.5% for the studied pH range. The results for TOC removal are similar and there is a high level of mineralization of MB (47–57%) in the studied pH range. The results show that NH_2_-MIL-88B(Fe) can effectively work in a wide pH range. This is different from the conventional Fenton reaction, which usually requires acidic conditions. This wide pH range can simplify the treatment procedure for practical application and lower the treatment cost. Therefore, the pH of the MB solution was not adjusted for the subsequent experiments.

The effect of temperature on the degradation efficiency of MB and TOC removal was also investigated. The degradation efficiency of MB and TOC removal are plotted against time at different temperatures in Figs 4d and S5 (Supporting Information). The degradation efficiency of MB and TOC removal are higher at higher temperature. Only 15 min is needed for complete removal of MB when the temperature is ≥308 K (Fig. 4d). This shows that the NH_2_-MIL-88B(Fe) MOF has high temperature tolerance because it can effectively work at a high temperature (318 K), and it is superior to most natural enzymes. The TOC abatement efficiency reaches 41.44% after 30 min reaction at room temperature (Fig. S5, Supporting Information). Hence, for operational convenience and practical consideration, room temperature was chosen for the following experiments.

### Possible catalytic mechanism

To determine the catalytic mechanism of NH_2_-MIL-88B(Fe), hydroxyl radical formation was investigated using terephthalic acid (TA) as a fluorescence probe. The hydroxyl radical can readily react with TA, forming highly fluorescent 2-hydroxyterephthalic acid^57^. As shown in Fig. S6 (Supporting Information), the fluorescence intensity of TA is weak in the absence of NH_2_-MIL-88B(Fe). However, it increases with increasing amount of NH_2_-MIL-88B(Fe). This suggests that ∙OH is generated by H_2_O_2_ decomposition. H_2_O_2_ decomposition accelerates as the amount of NH_2_-MIL-88B(Fe) increases and more hydroxyl radicals are generated, which was confirmed by ESR spectroscopy (Fig. 3b). The above observations confirm that the oxidation mechanism of MB catalyzed by NH_2_-MIL-88B(Fe) can be ascribed to generation of ∙OH by decomposition of H_2_O_2_. *p*-Benzoquinone (BQ) was also used to investigate the possible active intermediate in the reaction system, because it has been reported that BQ is a good trapper of O_2_^•−^ radicals^58^. The results indicate that MB degradation is inhibited in the presence of BQ (Fig. S7, Supporting Information), suggesting that O_2_^•−^ radicals are formed in catalytic degradation of MB by H_2_O_2_ in the presence of NH_2_-MIL-88B(Fe). To further confirm this, the effect of superoxide dismutase (SOD, the specific O_2_^•−^ scavenger) on the removal of MB was studied. As shown in Table S4, MB removal decreased by more than 28% in the presence of 20 U/mL SOD as the specific O_2_^•−^ scavenger, indicating the presence of O_2_^•−^ radical in MB removal.

### Recycling of NH_2_-MIL-88B(Fe)

**Figure 5:**
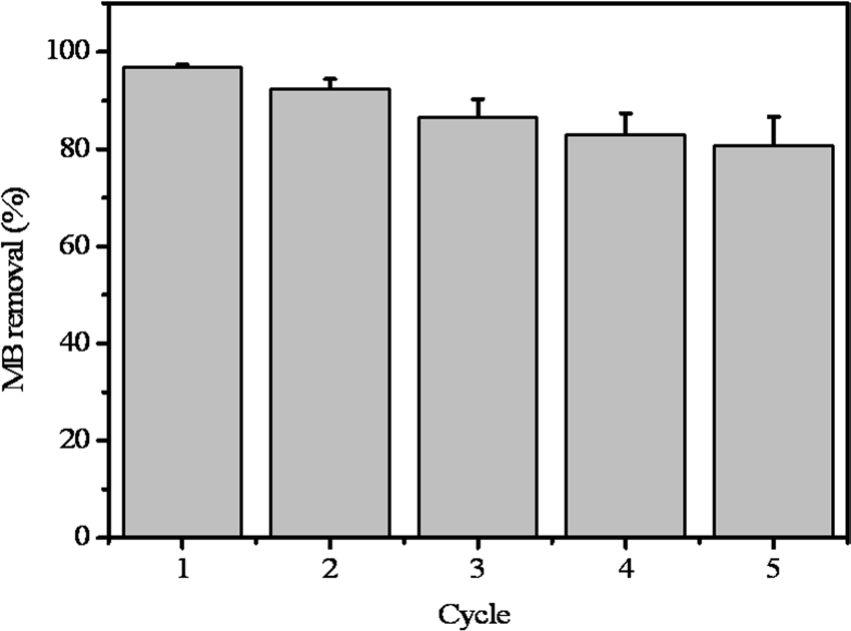
Removal efficiency of MB for different cycles of NH_2_-MIL-88B(Fe) using identical reaction conditions. Reaction conditions: pH 5.6, 0.3 g/L of NH_2_-MIL-88B(Fe), 0.2 M H_2_O_2_, 20 mg/L of MB. Error bars represent one standard deviation (*n* = 3).

When a MOF is used as a heterogeneous catalyst, its stability under the reaction conditions is an important issue that needs to be considered^10^. Hence, recycling experiments were performed to evaluate the durability of NH_2_-MIL-88B(Fe). The results show that MB removal is above 80% after five cycles (Fig. 5). The loss of the catalytic activity is probably related to the nanosize and good dispersibility of NH_2_-MIL-88B(Fe) in aqueous solution, leading to its partial recovery by centrifugation. The cumulative loss of Fe ions between the cycles is probably another reason for the observed decline in MB removal, although only a small amount of Fe^3+^ leaches out (<0.5%) at pH 3.0–11.0 and the concentration of dissolved iron in solution is less than 0.2 mg/L (Fig. S8, Supporting Information). In addition, after five cycles, the BET surface area of NH_2_-MIL-88B(Fe) decreased from 163.9 m^2^/g to 13.5 m^2^/g. The obvious decrease in the BET surface area suggests the collapse of NH_2_-MIL-88B(Fe) framework, leading to partial loss of crystallinity and cracks of the NH_2_-MIL-88B(Fe) (Figs S9 and S10, Supporting Information). This also contributes to the loss of catalytic activities of NH_2_-MIL-88B(Fe) after cycles. NH_2_-MIL-88B(Fe) can be easily separated from the reaction solution by simple centrifugation and reused. Thus, no additional chemicals are needed to regenerate NH_2_-MIL-88B(Fe), indicating that this MOF is a promising catalyst with potential applications in water purification.

## Conclusion

We have successfully synthesized the NH_2_-MIL-88B(Fe) MOF by a simple and facile microwave-assisted approach. The as-prepared NH_2_-MIL-88B(Fe) exhibits unique oxidase-like and peroxidase-like activities. We used the MOF as a mimic enzyme in the Fenton-like reaction for effective degradation of MB (a model toxic dye) in contaminated water. Complete removal of MB was achieved within 45 min. Regeneration studies show the feasibility of catalyst reuse. Importantly, removal of MB is above 80% after five cycles, showing the high stability of the as-prepared NH_2_-MIL-88B(Fe) MOF in aqueous media. Taking into account the easy preparation, wide pH tolerance, high efficiency, and good recycling ability, NH_2_-MIL-88B(Fe) is a promising Fenton-like catalyst for water purification.

## Experimental Section

### Chemicals and reagents

Iron(III) chloride hexahydrate (FeCl_3_·6H_2_O), 2-aminoterephthalic acid (NH_2_-BDC), TA, ethanol, *N*,*N*-dimethylformamide (DMF), MB, hydrogen peroxide, sodium hydroxide, and hydrochloric acid were purchased from Chongqing Taixin Chemical Co. Ltd. (Chongqing, China). TMB, *p*-benzoquinone, and 5,5-dimethyl-1-pyrroline *N*-oxide (DMPO) were obtained from Sigma-Aldrich (St. Louis, MO, USA) and stored in a refrigerator at 4 °C. All of the analytical reagents were used without further purification. All of the solutions were prepared using ultra-pure water. The solution pH was adjusted using dilute HCl and NaOH solutions.

### Preparation of the NH_2_-MIL-88B(Fe) MOF

NH_2_-MIL-88B(Fe) was prepared by the method reported by Ma *et al*.^29^ with slight modification. In brief, FeCl_3_·6H_2_O (124.5 mg, 0.46 mmol), NH_2_-BDC (83.4 mg, 0.23 mmol), and DMF (10 mL, 0.13 mol) were mixed by magnetic stirring for 30 min at room temperature to form a solution with a Fe^3+^/NH_2_-BDC/DMF molar ratio of 1:1:282. After the solution was degassed by shaking in an ultrasonic bath for 5 min, the resulting mixture was transferred into a 100 mL Teflon autoclave, which was sealed and placed in a microwave oven (Mars-5, CEM, maximum power 1200 W). The autoclave was heated to 150 °C in about 5 min and then maintained at this temperature for 15 min. The microwave power was set to 600 W throughout the whole synthesis process, including the heating-up stage. After the reaction, the resulting suspension was centrifuged. The obtained solid product was purified by triple treatment in DMF and ethanol at 60 °C for 1 h, and then dried under vacuum at 60 °C overnight. A reddish brown power was obtained with a yield of about 30%.

### Instrumentation

The ultraviolet–visible measurements were performed with a UV-2450 Shimadzu spectrophotometer (Suzhou, China). The SEM images were recorded by a Hitachi S-4800 field emission scanning electron microscope (Hitachi, Japan) with an accelerating voltage of 10 kV. The PXRD patterns of the as-prepared products were recorded with a XD-3X-ray diffractometer (PuXi, Beijing, China) under the conditions of nickel-filtered CuKα radiation (λ = 0.15406 nm) at a current of 20 mA and a voltage of 36 kV. The scanning rate was set to 4°/min. The specific surface area was determined by the Brunauer–Emmett–Teller method using an ASAP 2020 Micromeritics instrument (Maike, USA) at 77 K. The FTIR spectra were recorded with a Nicolet 170SX spectrometer (Madison, WI, USA) in transmission mode using KBr pellets of the sample. The TOC measurements were performed with a Hach IL TOC-550 TOC analyzer (Hach, USA). A MARS-5 microwave heating apparatus was used for preparation of NH_2_-MIL-88B(Fe). The ESR spectra were recorded with an X-band Bruker ESP300 E ESR spectrometer (Bruker, Germany).

### Catalytic removal experiments

The removal experiments were performed by adding 5 mg of NH_2_-MIL-88B(Fe) and H_2_O_2_ into 25 mL of 20 mg/L MB aqueous solution at room temperature for 45 min reaction time. The resulting mixture was then filtered through a 0.22 µm mixed fibre membrane (Tianjin Automatic Science Instrument Co., Ltd., Tianjin, China) and used for MB and TOC analysis. To evaluate the stability of the as-prepared NH_2_-MIL-88B(Fe) catalyst, after the removal experiment, the resulting mixture was centrifuged and the supernatant was discarded. The isolated NH_2_-MIL-88B(Fe) catalyst was directly used for the next cycle. The concentration of MB was determined by measuring the absorbance of the solution at 662 nm. The effect of the free radical inhibitor was evaluated by adding *p*-benzoquinone (an O_2_^•−^ radical quencher)^58^ into the reaction solution.

## Electronic supplementary material

Supporting Information
