# Supplementary material for: Highly efficient Fenton and enzyme-mimetic activities of NH2-MIL-88B(Fe) metal organic framework for methylene blue degradation
Source: Sci Rep. 2018 Mar 26;8:5159. doi: 10.1038/s41598-018-23557-2 (PMC5980107; doi:10.1038/s41598-018-23557-2)
Supplement: Supplementary file 1 — Supporting Information [file 41598_2018_23557_MOESM1_ESM.doc]

**Supporting Information**

**Highly efficient Fenton and enzyme-mimetic activities of NH_2_-MIL-88B(Fe) metal organic framework for methylene blue degradation**

Jianchuan He^1,2^ , Yao Zhang^1^, Xiaodan Zhang^1^, Yuming Huang^1^*

^1^The Key Laboratory of Eco-environments in Three Gorges Reservoir Region, Ministry of Education, College of Chemistry and Chemical Engineering, Southwest University, Chongqing 400715, PR China.

^2^School of Basic Medical Sciences, North Sichuan Medical College, Nanchong 637000, PR China.

*Corresponding authors. Tel./Fax: +86-23-68254346; E-mail addresses: yuminghuang2000@yahoo.com (Y. Huang)

**Supplemental Experimental**

**1. Adsorption kinetics**

Pseudo-first-order^1^ (eq 1) and pseudo-second-order^1^ (eq 2) were used to fit the experimental data and evaluate the adsorption kinetic process.

ln(*q*_e_ − *q*_t_ ) = ln *q*_e_ − *k*_1_ *t* (1)

*t*/*q*_t_ = 1/( *k*_2_ *q*_e_^2^) + *t*/*q*_e_ (2)

where *q*_e_ (mg/g) and *q*_t_ (mg/g) represent the adsorption capacity at equilibrium and at time *t*, respectively. *k*_1_ is the pseudo-first-order kinetic constant constant, and *k*_2_ is the pseudo-second-order kinetic constant (g mg^−1^ min^−1^). Table S1 shows the kinetic parameters of the two models.

**Table S1.** Pseudo-first-order and pseudo-second-order kinetic model parameters for MB adsorption on NH_2_-MIL-88B(Fe) MOF.

| pseudo-first-order | | pseudo-second-order | |
| --- | --- | --- | --- |
| *k*_1_  (min^-1^) | *R*^2^ | *k*_2_  (g mg^-1^ min^-1^) | *R*^2^ |
| 0.0828 | 0.8710 | 1.27×10^−3^ | 0.9940 |

**Table S2.** Pseudo-second-order rate constants (*k*_2_) of the Fe-MIL-88NH_2_ and MOF-235 for adsorption of MB.

| Adsorbents | *k*_2_ (g mg^-1^ min^-1^) | Refs |
| --- | --- | --- |
| MOF-235 | 9.58×10^-5^−2.18×10^-4^ | 5 |
| NH_2_-MIL-88B(Fe) | 1.27×10^−3^ | this work |

**2. Adsorption isotherms**

The Langmuir (eq 3)^1^ and Freundlich (eq 4)^3^ equations were employed to fit the experimental data:

*C*_e_/*q*_e_ = *C*_e_ /*q*_max_ + 1(/*q*_max_ *b*) (3)

log *q*_e_ = log *k*_f_ + (1/*n*) log *C*_e_  (4)

where *C*_e_ denotes the equilibrium concentration of the MB (mg/L), *q*_e_ is the equilibrium adsorption capacity (mg/g), *q*_max_ represents the maximal adsorption capacity of the adsorbent (mg/g), *b* is the Langmuir constant related to the energy of adsorption (L/mg), *k*_f_ and *n* are the constants of the Freundlich adsorption model correlated to the relative adsorption capacity and the adsorption intensity, respectively. The parameters calculated from these two models are listed in Table S3.

**Table S3**. Freundlich and Langmuir isotherm parameters for MB adsorption on NH_2_-MIL-88B(Fe)

| Freundlich | | | Langmuir | | |
| --- | --- | --- | --- | --- | --- |
| *n* | *k*_f_ | *R*^2^ | *q*_max_ | *b* | *R*^2^ |
| 5.81 | 32.84 | 0.6568 | 61.46 | 0.59 | 0.9987 |

**3. Hydroxyl radical formation with terephthalic acid as a fluorescence probe**

50 mM H_2_O_2_, 0.6 mM terephthalic acid and different concentrations of the NH_2_-MIL-88B(Fe) were first incubated at 25 °C for 10 min, then the NH_2_-MIL-88B(Fe) was removed from the reaction solution by an centrifugation. The supernatant was used for fluorometric measurement. Hydroxyl radical can react readily with terephthalic acid (TA), forming highly fluorescent 2-hydroxy terephthalic acid^4^ that can be identified from fluorescence spectrometer. As shown in **Figure S6**, the fluorescence intensity of H_2_O_2_-TA system was weak in the absence of NH_2_-MIL-88B(Fe). However, the gradual increase of the fluorescence intensity was observed as the concentration of the NH_2_-MIL-88B(Fe) increased, indicating that the amount of the generated ·OH increased by the catalysis of NH_2_-MIL-88B(Fe).

***References***

1. E. Haque, J. E. Lee, I. T. Jang, Y. K. Hwang, J. S. Chang, J. Jegal, S. H. Jhung, *J. Hazard. Mater.,* 2010, **181**, 535−542.
2. J. W. Jun, M. Tong, B. K. Jung, Z. Hasan, C. Zhong and S. H. Jhung, *Chem. Eur. J.,* 2015, **21**, 347−354.
3. S. Jiaa, Y. Zhang, Y. Liu, F. Qin, H. Ren, S. Wu, *J. Hazard. Mater.,* 2013, **262**, 589– 597.
4. K. Ishibashi, A. Fujishima, T. Watanabe and K. Hashimoto, *J. Photoch. Photobio. A,* 2000, **134**, 139–142.
5. E. Haque, J. Jun, S. H. Jhung, *J. Hazard. Mater.,* 2011, **185**, 507– 511.

**Table S4.** Effects of SOD on the removal of MB (Reaction conditions: 25 °C, pH 5.6, 20 min, 0.2 g/L NH_2_-MIL-88B(Fe), 0.2 M H_2_O_2_, 20 mg/L MB solution)

| scavengers | intermediates | concentration | MB removal  (%) | percent inhibition  (%) |
| --- | --- | --- | --- | --- |
| / | / | / | 83.94 | / |
| SOD | O_2_^•−^ | 20 U/mL | 60.38 | 28.07 |

**Figure S1.** The FT-IR spectra of NH_2_-MIL-88B(Fe).

**Figure S2.** Adsorption kinetics of MB adsorption on NH_2_-MIL-88B(Fe). Reaction conditions: 5 mg NH_2_-MIL-88B(Fe), 25 mL of 20 mg/L MB solution, temperature 25 °C.

**Figure S3.** Adsorption isotherm for adsorption of MB on NH_2_-MIL-88B(Fe). Reaction conditions: 5 mg NH_2_-MIL-88B(Fe), 25 mL of MB solution with different concentrations, contact time 1 h, temperature 25 °C.

**Figure S4.** Variation of the zeta-potential of NH_2_-MIL-88B(Fe) under different pH value.

**Figure S5**. Effect of reaction temperature on the removal of TOC ( pH 5.6, H_2_O_2_ concentration: 0.2 M; concentration of initial MB: 20 mg/L).

**Figure S6**. The effect of NH_2_-MIL-88B(Fe) concentration on the formation of hydroxyl radicals with terephthalic acid (TA) as a fluorescence probe. Reaction conditions: TA concentration: 0.6 mM; H_2_O_2_ concentration: 50 mM; reaction temperature: 25 °C; reaction time: 10 min.

**Figure S7**. Effect of BQ on the degradation of MB. Reaction conditions: pH 5.6; reaction temperature: 25 °C; 0.2 g/L of NH_2_-MIL-88B(Fe); 0.2 M of H_2_O_2_; 20 mg/L of MB. Error bars represent 1 standard deviation for three measurements.

**Figure S8**. Leaching of Fe ions (black line) and dissolved iron concentration (blue line) after suspension of 5 mg of NH_2_-MIL-88B(Fe) in 25 mL of water with different pH values. Error bars represent 1 standard deviation for three measurements.

**Figure S9**. The PXRD patterns of NH_2_-MIL-88B(Fe) before (a) and after (b) catalytic experiment.


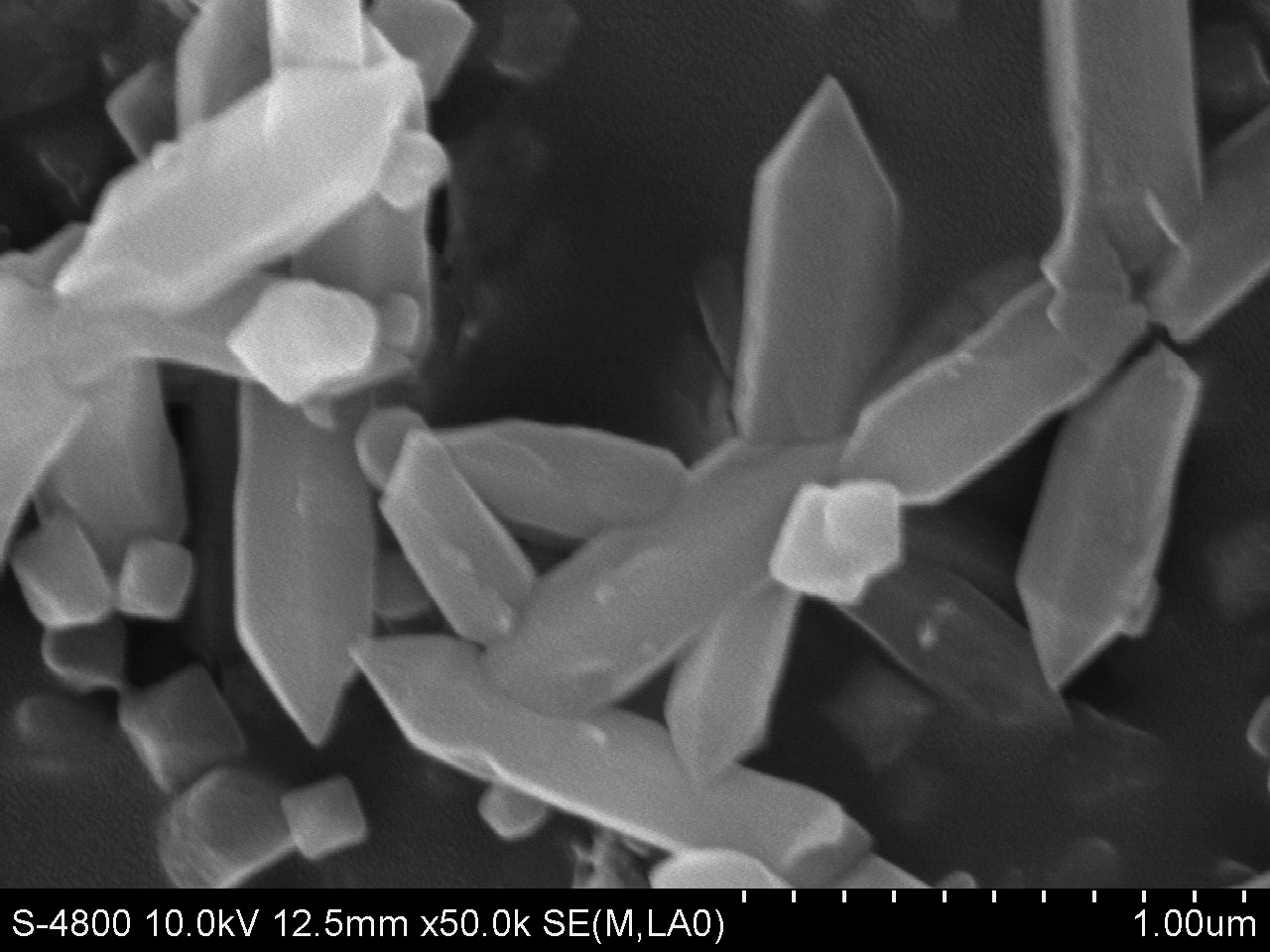

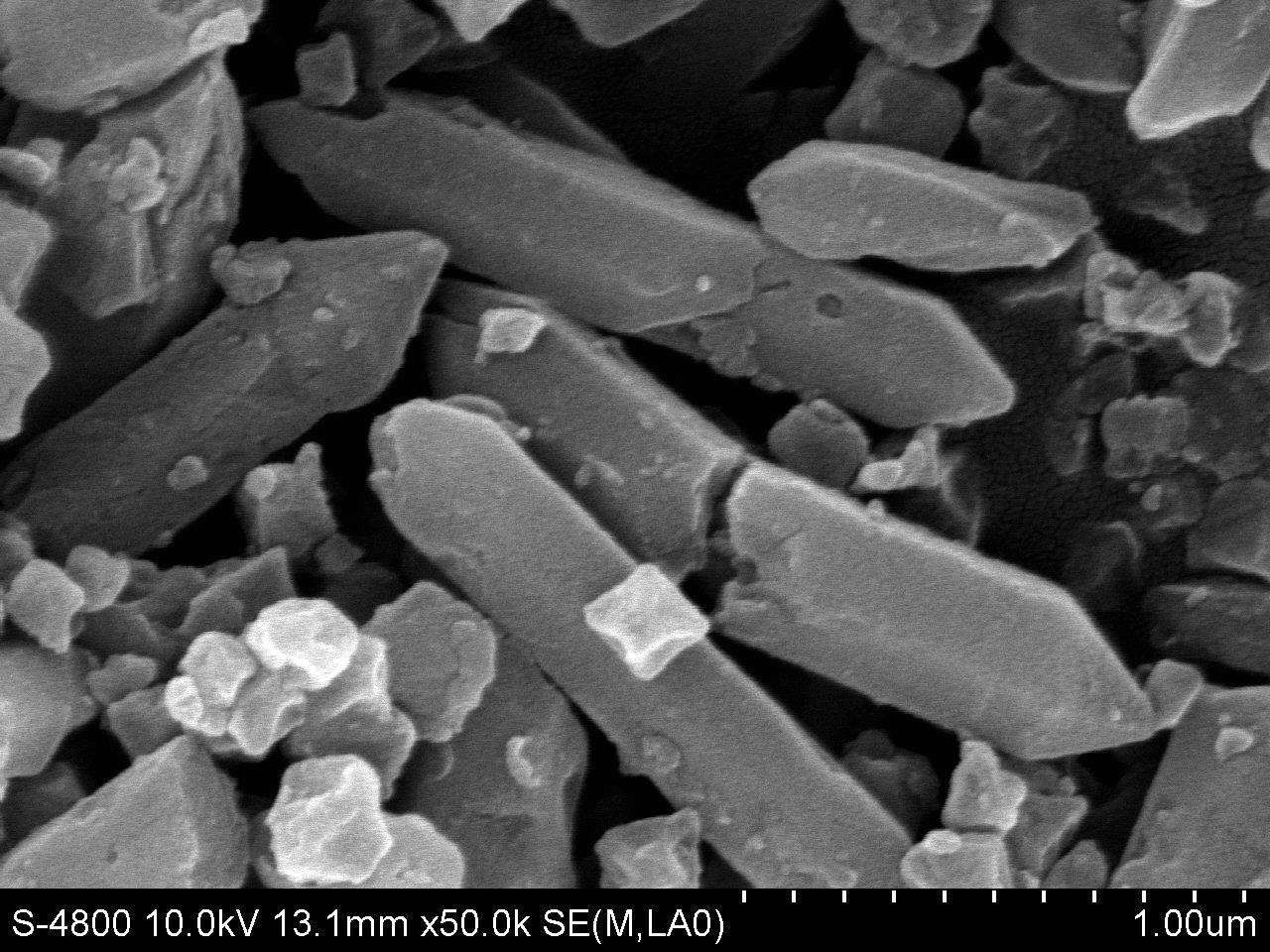


(a) (b)

**Figure S10**. The SEM images of NH_2_-MIL-88B(Fe) before (a) and after (b) catalytic experiment.
